# Mechanical Complication with Broviac Repair Kit in a 4-Year-Old Boy with MEN 2a

**DOI:** 10.1155/2009/693583

**Published:** 2009-06-23

**Authors:** Sergio B. Sesia, Frank-Martin Haecker, Johannes Mayr

**Affiliations:** Department of Pediatric Surgery, University Children's Hospital Basle (UKBB), 4005 Basle, Switzerland

## Abstract

*Background*. Mechanical complications in the use of indwelling central venous catheters (CVCs) such as the Broviac catheter (BC) include kinking, occlusion, dislocation or leaking. We report on a mechanical complication after using a repair kit for the BC. *Method*. A 4-year old boy, suffering from multiple endocrine neoplasia type 2a (MEN 2a), intestinal aganglionosis (Hirschsprung's disease), and short bowel syndrome, required a BC for home parenteral nutrition. *Result*. Due to recurrent leakage of the BC, 5 subsequent repairs were necessary within seven months. During one repair a metallic tube belonging to the repair kit was found to have migrated proximally to the skin entrance level within the BC and requiring surgical removal. *Conclusion*. To our knowledge, this is the first report focusing on such a serious complication using a BC and its repair kit. The proximal migration of this metallic tube constitutes a distinct theoretical risk of endothoracic foreign body embolization.

## 1. Background

**Figure 1:**
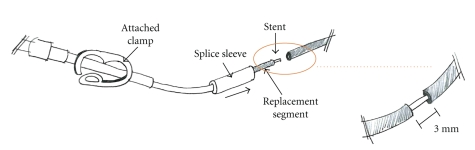
Attachment of external segment.

**Figure 2:**
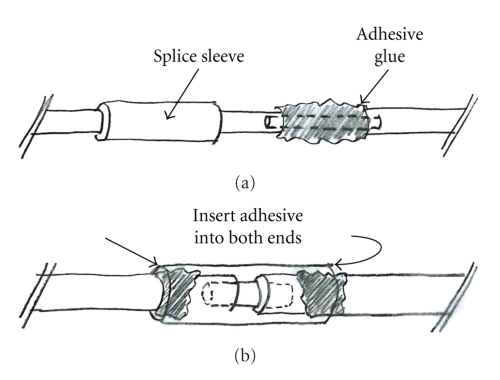
Application of adhesive onto the outside of the stent and cover of both ends of catheters by a splice sleeve, fixed with additional adhesive.

 Indwelling central venous catheters (CVCs) such as the Broviac^®^ catheter (BC) offer reliable vascular access and are indispensable for long-term chemotherapy in cancer patients or patients undertaking long term parenteral nutrition [1]. However, several disadvantages of the application of CVCs have been reported [2]. Possible complications include infection [3], thrombosis [4] or mechanical problems [5] such as dislocation or leaking caused by repeated bending of the external segment or clamping maneuvers. When such problems occur the patency of the occluded CVC-lumen can be restored via intraluminal installation of streptokinase [6]. Also special repair kits (Bard, Bard Access Systems, reference 0601610CE, Salt Lake City, Utah, USA) allow repair of the damaged BC and spare the need for its reimplantation. After cutting the external portion of the damaged catheter distal to the broken area. The stent premounted into the replacement catheter segment is inserted into the indicated catheter lumen (Figure 1). Then, adhesive is applied to the outside of the stent and both ends of the catheter are covered by a splice sleeve and fixed with additional adhesive (Figure 2). The CVCs used in children for long term venous access are usually tunneled subcutaneously. We report on a potentially serious mechanical complication using a Broviac^®^ repair kit in a boy suffering from MEN 2a syndrome and long-segment Hirschsprung's disease. To our knowledge, this represents the first report of this type of mechanical complication with this catheter repair kit. 

## 2. Case Report

**Figure 3:**
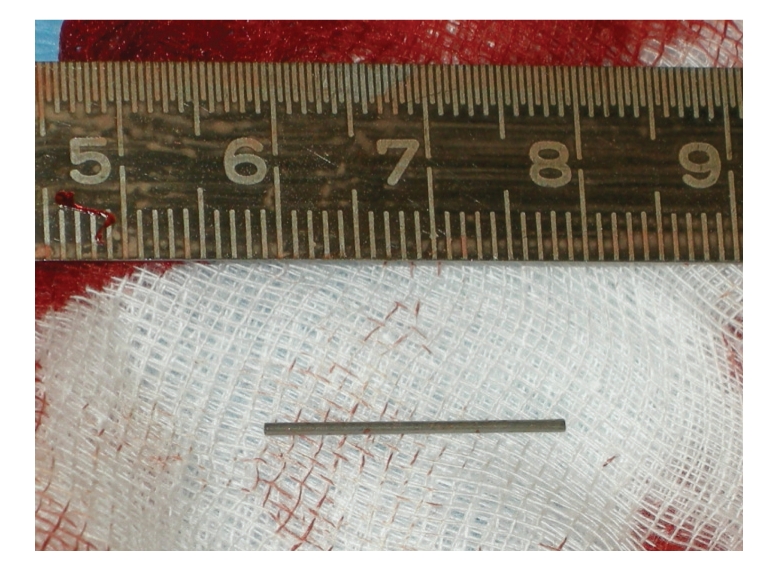
Photo of removed metallic tube.

**Figure 4:**
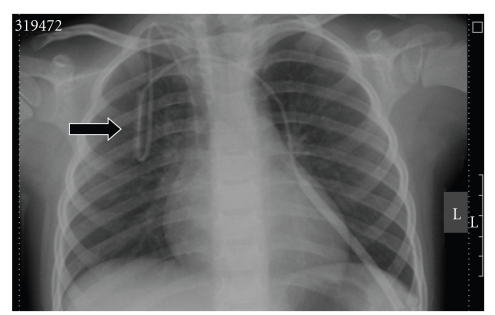
X-ray with correct position of the CVC, repaired area indicated by arrow.

 A 4-year old boy suffering from MEN 2a, long segment Hirschsprung's disease (total colonic aganglionosis), short bowel syndrome, von Willebrand's disease type 1, heparin-induced thrombopenia (HIT) type 2, and psychomotoric retardation is documented in this case study. A C618G mutation in exon 10 of the RET gene was demonstrated in DNA extracted from blood. Due to weight loss a French 4.2 BC catheter (Bard^®^, Bard Access Systems, reference 0600060CE, Salt Lake City, Utah, USA) had been inserted 15 months previously to allow administration of home parenteral nutrition by infusion pump (Abbott Laboratories GEMSTAR, Reference 13000-15-05/06, North Chicago, Ill, USA) 14 hours a day. Because of the occurrence of HIT in this patient the BC was blocked each morning using lepirudin. Three months before the described event occurred, the BC had to be replaced surgically because of thrombus formation in the superior vena cava adjacent to the tip of the BC. An attempt at inducing thrombolysis using Urokinase proved to be ineffective. Due to the infant's activity and repeated clampings at the clamping site, leaks of the external BC segment had been repaired twice within the last 3 months using the commercial BC repair kit (Bard^®^, Bard Access Systems, reference 0601610CE, Salt Lake City, Utah, USA). An X-ray taken after the previous repair demonstrated the correct position of the CVC (Figure 4). Seven weeks after the last repair, malfunction of the catheter with increased resistance when applying the home parenteral nutrition solution was noted by outpatient nurses and the patient's mother at home. During inspection of the former BC repair region, we noticed a missing central metallic tube (MT, Figure 3), together with a transparent appearing repair site area. By gentle palpation of the proximal BC, migration of the central MT was suspected, and a palpable resistance within the BC at the skin entrance level was noted. By gentle milking maneuvers the MT was manipulated distally and the MT was removed after transection of the BC 5 cm above skin level. Another repair kit for Broviac French 4.2 catheters was used to repair the transected BC.

## 3. Discussion

As described in literature [6], the most common mechanical complication reported to be associated with the use of the BC is occlusion. In the current case repeated BC leakage required replacement of the external segment of the BC using a commercial repair kit which included a metallic tube to assure the connection between the old and the new portion of the BC. This metallic tube obviously became loose and migrated proximal to the skin entrance site of the BC. This mechanical complication can easily be detected by a caregiver or medical staff member, due to the completely transparent appearance of the repaired area of the BC, indicating that the metallic tube (Figure 3) has migrated away from the repaired area. In the case that the missing MT can be palpated within the catheter, a milking maneuver may be performed to prevent the need for central replacement of the BC. This maneuver allows the manipulation of the metallic tube into its distal position in order to gain enough length and to perform another BC repair procedure. In the case that the central MT cannot be felt by gentle palpation of the BC, we recommend to perform an X-ray examination of the thorax and neck, in order to localize the metallic tube. If the metallic tube has migrated beneath the skin level but it is still located within the BC, removal and replacement of the BC should be the treatment of choice. In order to avoid degradation of the material consistence on the repaired area (reduced adherence and modified texture of the BC), at each replacement we routinely resected the damaged area. However, the risk of dislocation of the MT may be greater than if repair is performed only once. Due to the frequently observed kinking of the BC at the entrance site into the vessel it seems unlikely that embolisation of the metallic tube will occur. On the other hand, the occurrence of this potentially dangerous complication cannot be excluded with certainty, and removal of the metallic tube from inside the heart or pulmonary vessels can be a hazardous endovascular or cardiothoracic procedure.

## Abbreviations

CVC: Central venous catheter
BC: Broviac^®^ catheter
MT: Metallic tube.
